# Discordance of PIK3CA mutational status between primary and metastatic breast cancer: a systematic review and meta-analysis

**DOI:** 10.1007/s10549-023-07010-1

**Published:** 2023-07-01

**Authors:** Justus Rosin, Ella Svegrup, Antonios Valachis, Ioannis Zerdes

**Affiliations:** 1 School of Medical Sciences, Örebro University, Örebro, Sweden; 2 Department of Oncology, Faculty of Medicine and Health, Örebro University Hospital, Örebro University, Örebro, Sweden; 3 Department of Oncology-Pathology, Karolinska Institutet, Stockholm, Sweden; 4 Breast Center, Theme Cancer, Karolinska University Hospital & Karolinska Comprehensive Cancer Center, Stockholm, Sweden

**Keywords:** PIK3CA, Breast cancer, Mutation, Primary, Metastasis

## Abstract

**Introduction:**

In light of the clinically meaningful results of the PI3K inhibitors in *PIK3CA*-mutated metastatic breast cancer (BC) patients, the reliable identification of *PIK3CA* mutations is of outmost importance. However, lack of evidence on the optimal site and timing of assessment, presence of temporal heterogeneity and analytical factors pose several challenges in clinical routine. We aimed to study the discordance rates of *PIK3CA* mutational status between primary and matched metastatic tumors.

**Methods:**

A systematic literature search was performed in three different databases (Embase, Pubmed, Web of Science) and—upon screening—a total of 25 studies reporting *PIK3CA* mutational status both on primary breast tumors and their matched metastases were included in this meta-analysis. The random-effects model was used for pooled analyses of discordance of *PIK3CA* mutational status.

**Results:**

The overall discordance rate of *PIK3CA* mutational status was 9.8% (95% CI, 7.0–13.0; *n* = 1425) and did not significantly differ within BC subtypes or metastatic sites. The change was bi-directional, more commonly observed from *PIK3CA* mutated to wild-type status (14.9%, 95% CI 11.8–18.2; n tumor pairs = 453) rather than the opposite direction (8.9%, 95% CI 6.1–12.1; n tumor pairs = 943).

**Conclusions:**

Our results indicate the need of obtaining metastatic biopsies for *PIK3CA*-mutation analysis and the possibility of testing of the primary tumor, in case a re-biopsy deemed non-feasible.

**Supplementary Information:**

The online version contains supplementary material available at 10.1007/s10549-023-07010-1.

## Introduction

Despite the therapeutic advances in the management of breast cancer (BC), metastatic disease still remains incurable. However, the identification of molecular alterations has led to the development of novel targeted treatments which substantially prolong survival outcomes in the advanced setting.

The phosphatidylinositol 3-kinase (PI3K)/protein kinase B (AKT)/mammalian target of rapamycin (mTOR) represents one of the signaling pathways which plays crucial role in cell proliferation, growth and other cellular processes in several cancer types, including BC and more prominently the hormone receptor positive (HR +)/human epidermal growth factor receptor-2 negative (HER2-) subtype [1]. The pathway hyperactivation occurs mainly due to oncogenic mutations in *PIK3CA* gene encoding the p110a catalytic subunit of the PI3Kα heterodimeric protein complex, observed in approximately 40% of HR + /HER2- BC patients [2]. *PIK3CA* mutations display differential frequency and prognostic value in the early and metastatic setting whereas they have been associated with resistance to endocrine and HER2-targeted treatment [3–5]. Of note, inhibition of PI3K/Akt/mTOR pathway has provided clinically meaningful improved outcomes, mostly in patients with HR + /HER2- metastatic disease who have developed endocrine resistance [6, 7]. Based on randomized evidence showing significant progression-free and numerical clinically meaningful overall survival benefit when the oral PI3K—selective inhibitor alpelisib is combined with fulvestrant in patients with *PIK3CA*-mutated HR + /HER2- advanced or metastatic BC who progressed during or after endocrine therapy, this treatment combination has been approved from regulatory authorities [8, 9].

The predictive role of *PIK3CA* mutational status for the treatment with alpelisib poses some challenges on how evidence from randomized trials is implemented into clinical practice. The detection of *PIK3CA* mutations in archival or fresh tumor tissue and plasma-derived circulating tumor DNA (ctDNA) is dependent on analytical and methodological factors, thus possibly affecting clinical validity and utility [10]. Furthermore, the presence of tumor heterogeneity and clonal evolution over time could potentially influence the mutational status and any discordance between primary and metastatic disease should be acknowledged and managed based on available evidence. Following the paradigm of other common BC biomarkers (i.e. ER, PR, HER2, PD-L1), little is known about how *PIK3CA* mutational status would change during metastatic progression and potentially drive treatment selection in BC patients.

Given the treatment option of PI3K inhibitors in patients with HR + /HER2- metastatic breast cancer, the temporal heterogeneity within breast tumors and the diagnostic challenges associated with identification of mutational status, reliable identification of *PIK3CA*-mutated patients who will benefit from treatment with PI3K inhibitors remains of outmost importance. The aim of this systematic review and study-level meta-analysis was to evaluate the discordance rates of *PIK3CA* mutational status between primary and matched metastatic tumors in BC patients.

## Methods

### Search algorithm and study selection criteria

The protocol of the current systematic review and meta-analysis has been published on the PROSPERO database (CRD42023398005).

The literature search was performed in the following three databases: PubMed and Web of Science (November 2022) and Embase (February 2023). The detailed search strategy is presented in the Supplementary Material and included terms “PIK3CA”, “breast cancer” and “primary or metastatic disease” (MeSH terms) in the title or abstract. These terms were also adapted according to the corresponding Embase controlled vocabulary.

Studies were included in the meta-analysis based on the following criteria: (i) studies reporting *PIK3CA* mutational status both on primary breast tumors and their matched metastases, using tumor tissue, irrespective of the detection method; (ii) studies that included at least 10 patients. Studies including circulating tumor cells or ctDNA analyses, in vitro and/or in vivo experiments, reviews, case reports or previous meta-analyses or written in language other than English were excluded.

### Data extraction and quality assessment

The initial study selection on the basis of abstract and title screening and the full text screening were performed independently by two investigators (JR, ES), a third investigator (AV) resolved any discrepancies and consensus was reached for all eligible studies. Two investigators (JR, ES) performed the data extraction using a predefined form and a third (IZ) resolved any discrepancies by comparing the databases.

The following data were collected from each study: first author, journal, year of publication, country, if a study was multicentric or if only a single center was involved, study type (prospective/retrospective), number of patients with paired samples, if the study focused on specific metastatic sites and if it concerned a specific breast cancer subtype, overall number of discordant cases, direction of discordance (from *PIK3CA-*mutated in primary tumor to *PIK3CA*-wild-type in metastasis or vice versa) and mutation detection method.

The reporting quality of all studies included in the meta-analysis were assessed by two investigators (JR, ES) and a third investigator resolved any discrepancies, according to REporting recommendations for tumour MARKer prognostic studies (REMARK) checklist, consisting of 20 items, as previously described [11]. Each item (involving the reporting of study aims, methods, results and their contextual discussion) was scored on a scale from 0 to 2 depending on how adequately each of the 20 items was defined in the study, thus generating a maximum total quality assessment score of 40.

### Outcomes and definitions

The outcomes of interests included: i) overall discordance (n pairs with discordance / total n of pairs analyzed), ii) overall discordance rate from *PIK3CA*-mutated to *PIK3CA*-wild-type (n samples changed from mutated to wild-type in metastatic tumor / n samples with *PIK3CA*-mutated in primary tumor), iii) overall discordance rate from *PIK3CA*-wild-type to *PIK3CA*-mutated (n samples changed from wild-type to mutated in metastatic tumor / n samples with *PIK3CA-*wild-type in primary tumor).

### Statistical analysis

The discordance rates were calculated for the overall population and when feasible, within breast cancer subtypes (HR + /HER2-negative, HER2-positive, triple negative breast cancer), according to the site of recurrence (locoregional / distant) or per metastatic site (brain / liver / other).

The random-effects model was used for pooled analyses of discordance and corresponding 95% confidence interval (CI). Chi-square test was used to compare potential differences among the pooled discordant rates. Sensitivity analysis was performed within subgroups of interest, by excluding studies either without HER2 status or using gene expression profiling data for defining subtypes. Publication bias was evaluated based on both the visual inspection/qualitative assessment of the asymmetry on funnel plots and Egger’s test. Statistical heterogeneity was assessed using I^2^ statistic for each pooled analysis, with a 50% cut off for considerable heterogeneity. Each reported p-value was two-sided with significance being set at 0.05. All statistical analyzes were performed with StatsDirect (StatsDirect Ltd. UK, 2013).

## Results

### Study characteristics

**Fig. 1:**
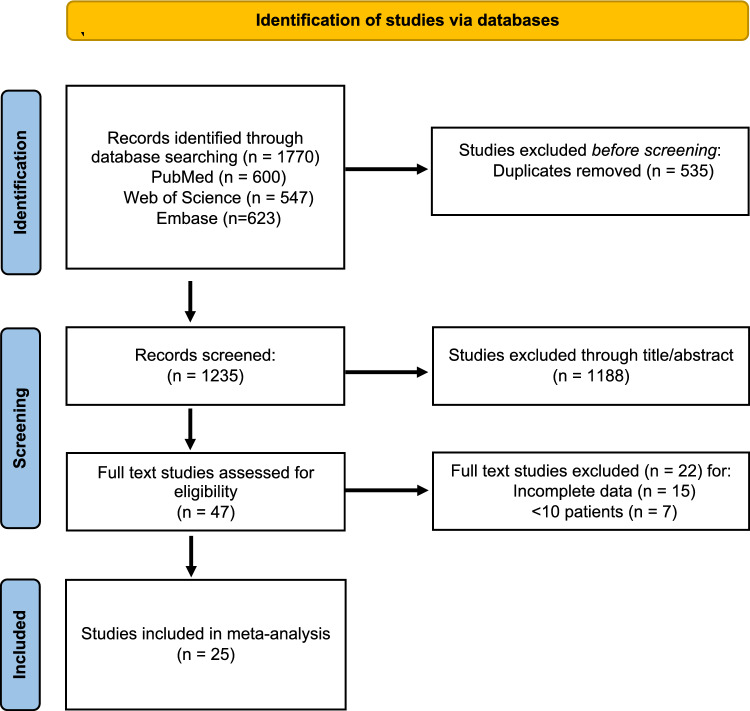
PRISMA flowchart of search and study selection

The Preferred Reporting Items for Systematic Reviews and Meta-analyses (PRISMA) study flowchart is depicted in Fig. 1. The initial search generated a total of 1770 records, 600 from PubMed, 547 from Web of Science and 623 from Embase. Upon deduplication, 1235 studies were screened for abstract and title 47 were assessed in full text.; 25 studies fulfilled the criteria and included in the meta-analysis.

The studies were published between 2010 and 2022 and the study characteristics are summarized in Table 1 [12–36]. The number of paired cases ranged between 10 and 242 patients. Twenty-one out of the 25 studies (84%) included in the meta-analysis were retrospective whereas the rest included patient cohorts collected within the scope of a prospective study. None of the studies presented results on treatment outcomes with alpelisib or other PI3K inhibitors. *PIK3CA* mutations were detected using targeted next-generation sequencing [21] methodology in the majority of the studies (14 of 25; 56%). A total of 19 studies (76%) evaluated the PIK3CA mutational status according to the site of recurrence (locoregional and/or distant), while 13 studies (52%) focused on specific metastatic sites including brain, liver or other localization (Table 2).

**Table 1:** Characteristics of the studies included in the meta-analysis

Author (reference)	Year	Country	Study type	Multi-center study	Metastatic site	Number of paired patient samples	Detection method	Quality assessment score
Aftimos (12)	2021	Belgium	Prospective	Yes	All	242	tNGS	34
Agahozo (13)	2019	Netherlands	Retrospective	No	All	26	SNaPshot, dPCR	17
Akahane (14)	2020	Japan	Retrospective	Yes	All	11	tNGS	19
Akcakanat (15)	2021	USA	Retrospective	No	All	10	tNGS	23
Arthur (16)	2014	UK	Retrospective	No	All	89	PCR	28
Basho (17)	2016	USA	Retrospective	Yes	All	89	tNGS	29
Bertucci (18)	2016	France	Retrospective	No	All	23	aCGH, tNGS	20
Callens (19)	2021	France	Prospective	Yes	All	67	tNGS, WES	30
Chen (20)	2021	China	Retrospective	No	Lymph nodes	131	tNGS	22
Da Silva (21)	2010	Australia	Retrospective	Yes	Brain	12	PCR combined with MALDI-TOF MS	18
Drury (22)	2011	UK	Retrospective	Yes	All	21	PCR	22
Dupont Jensen (23)	2011	Denmark	Retrospective	No	All	100	SNaPshot, RT-PCR	29
Fumagalli C (24)	2020	Italy	Retrospective	No	All	61	tNGS	29
Fumagalli D (25)	2016	Belgium	Prospective	No	All	68	PCR-based MUT-MAP	31
Giannoudis (26)	2021	UK	Retrospective	No	Brain	32	PCR-based UltraSEEK® panel	19
Gonzalez-Angulo (27)	2011	USA	Retrospective	Yes	All	47	PCR- and mass spectometry based	21
Gonzales-Martinez (28)	2022	Spain	Retrospective	Yes	Skin	33	tNGS	25
Kim (29)	2019	South Korea	Retrospective	Yes	All	19	PCR combined with MALDI-TOF MS	36
Lee (30)	2015	South Korea	Retrospective	No	Brain	15	tNGS	20
Meric-Bernstam (31)	2014	USA	Retrospective	Yes	All	33	tNGS	20
Park (32)	2022	South Korea	Retrospective	No	All	49	ddPCR	23
Roy-Chowduri (33)	2015	USA	Prospective	No	All	31	tNGS	23
Schleifman (34)	2014	USA	Retrospective	No	All	73	PCR-based MUT-MAP, SNP	21
Thulin (35)	2021	Sweden	Retrospective	Yes	Brain	37	tNGS	24
van Geelen (36)	2020	Australia	Prospective	No	All	76	tNGS	30

*tNGS* targeted next-generation sequencing, *dPCR* digital polymerase chain reaction, *RT-PCR* real-time polymerase chain reaction, *aCGH* array-based comparative genomic hybridization, *WES* whole-exome sequencing; *SNaPshot genotyping* primerextension or minisequencing, *ddPCR* droplet digital polymerase chain reaction, *MUT-MAP* mutation multi-analyte panel, *SNP* single nucleotide polymorphism genotyping, *MALDI-TOF MS* matrix-assisted laser desorption/ionization coupled to time-of-flight mass spectrometry

**Table 2:** Pooled discordance rates according to direction of *PIK3CA* mutational status change and within subgroups of interest

Parameters	N studies (*n* pairs)	Pooled discordance, %	95% Confidence Interval, %	Statistical heterogeneity (I^2^)	p-value for comparison
*Direction of change*					0.003
Mut to wild-type	24 (453)	14.9	11.8–18.2	35.3	
Wild type to mut	24 (943)	8.9	6.1–12.1	56.4	
*Breast cancer subtype*					0.577
HR + /HER2-negative	13 (583)	10.2	6.4–14.8	56.6	
HER2-positive	10 (149)	8.7	4.8–13.7	0.0	
TNBC	11 (151)	8.8	4.9–13.7	0.0	
*Site of recurrence*					0.839
Locoregional	8 (306)	9.6	6.6–13.1	29.0	
Distant (any)	11 (301)	9.9	6.8–13.5	49.4	
*Metastatic site*					0.330
Brain	5 (106)	9.6	1.1–24.9	77.2	
Liver	3 (59)	6.4	1.7–13.9	0.0	
Other	5 (67)	11.2	4.9–19.6	7.2	

### Quality of the eligible studies, magnitude of heterogeneity and publication bias

The median reporting quality assessment score was 23 (range: 17–36) according to REMARK guidelines. Substantial statistical heterogeneity among eligible studies (I^2^ > 50%) was observed in most of the analyses, except for the overall discordance rate of *PIK3CA* mutational status from mutated to wild-type and the HER2 + and triple-negative breast cancer (TNBC) subgroup analyses. Furthermore, no convincing evidence of publication bias was observed in pooled analyses (Supplementary Fig. 1).

### Pooled discordance rates of PIK3CA mutational status and direction of change in paired primary and metastatic tumors

**Fig. 2:**
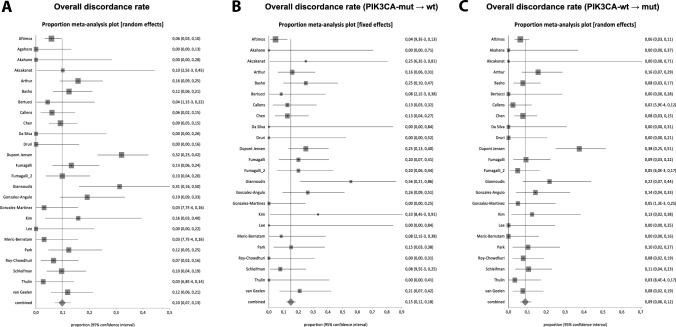
Forest plots for *PIK3CA* mutational status pooled discordance rates. **A** Overall discordance rate. **B** Overall discordance rate from *PIK3CA*-mutated to *PIK3CA*-wild-type. **C** Overall discordance rate from *PIK3CA*-wild-type to *PIK3CA*-mutated

The overall discordance rate of *PIK3CA* mutational status between matched primary and metastatic breast cancer, regardless of subtype was 9.8% (95% CI, 7.0–13.0; I^2^ = 70.0%), including 1425 patients from 25 studies (Fig. 2A). The direction of change was more commonly observed from *PIK3CA* mutated to wild-type status (14.9%, 95% CI 11.8–18.2; n studies = 24, n tumor pairs = 453; I^2^ = 35.3%) (Fig. 2B) rather than from *PIK3CA* wild-type to mutated status (8.9%, 95% CI 6.1–12.1; n studies = 24; n tumor pairs = 943; I^2^ = 56.4%) (Fig. 2C). The difference between the directions of *PIK3CA* mutational status change was statistically significant (p = 0.003) (Table 2).

### Pooled discordance rates of PIK3CA mutational status in paired primary and metastatic lesions in subgroups of interest

Pooled results of *PIK3CA* mutational status discordance between matched primary and metastatic breast tumors within the different breast cancer subtypes of interest are presented in Table 2. Data from 13 studies including 583 HR + /HER2- BC patients demonstrated an overall discordance of 10.2% (95% CI 6.4–14.8; I^2^ = 56.6%). A sensitivity analysis was performed after exclusion of studies without available HER2 status or with subtyping based on gene expression profiling data and showed similar pooled discrepancy rates (9.7%, 95% CI 5.1 – 15.6%, n = 442 and 10.3%, 95% CI 5.8 – 15.8%, n = 515, respectively). A comparable overall discordance rate was noted between HER2-positive (8.7% 95% CI 4.8–13.7, *n* = 149, I^2^ = 0%) and TNBC (8.8%, 95% CI 4.9–13.7, *n* = 151, I^2^ = 0%) breast cancer patients (*p* = 0.557 for the comparison among the three subtypes). Similar results on the discrepancy rates were obtained though sensitivity analyses, upon exclusion of gene expression-based studies for both subtypes (8.8%, 95% CI: 4.6–14.0%, *n* = 133 for HER2 + and 8.2%, 95% CI: 4.0–13.8%, *n* = 117 for the TNBC).

Further subgroup analyses were performed according to the site of recurrence and metastatic site. Comparable pooled discordance rates were noted between the primary tumors and matched locoregional (9.6%, 95% CI 6.6–13.1) or distant (9.9%, 95% CI 6.6–13.1) recurrences (*p* = 0.839 for the comparison). When discordance rates were pooled based on distant metastatic site, a numerical but not statistically significant difference was observed between patients with matched metastatic lesion from brain compared to liver (9.6% versus 6.4%) (Table 2).

## Discussion

This is, to the best of our knowledge, the first meta-analysis providing data on the discordance rates of *PIK3CA*-mutations between primary breast tumors and their matched metastases. Given the clinically meaningful results of the PI3K inhibitors in *PIK3CA*-mutated metastatic BC patients [8, 9], the reliable detection of *PIK3CA* mutations remains of outmost importance as it could guide physicians’ choices (level of evidence I-A according to ESMO Scale for Clinical Actionability of Molecular Targets (ESCAT) score) [37]. The results of our meta-analysis indicate that the pooled discordance rate of *PIK3CA* mutations between primary tumors and paired metastases was relatively low, observed in approximately 1 out of 10 patients. Of note, we also showed that the discordance of *PIK3CA* mutational status was bi-directional, though more commonly observed from *PIK3CA*-mutated in primary tumor to wild-type status in the metastases rather than in the opposite direction. This finding could help clinicians in deciding how to proceed with *PIK3CA*-mutation analysis in clinical practice to ensure reliable results and thus facilitating decision-making process.

The prevalence and clinical implications of *PIK3CA* mutations in BC varies according to the disease setting and also within subtypes. In early BC, *PIK3CA* mutations have been detected in 37%, 22% and 18% of ER + /HER2-, HER2 + and ER-/HER2- subtypes, respectively and associated with improved invasive disease-free survival but also with resistance to HER2-targeted treatments [3, 5]. In contrast, *PIK3CA* mutations have been detected in 28% of HR + /HER2- metastatic BC patients and correlated with worse overall survival as well as resistance to chemo- and endocrine therapy [4]. However, none of the aforementioned studies have assessed *PIK3CA* mutations in matched primary and metastatic tumors. When evaluating the discordance rates in paired samples in the present study, we demonstrated comparable results among all different BC subtypes.

In the pivotal phase III randomized trial SOLAR-1 that confirmed the predictive value of *PIK3CA*-mutation status on the clinical benefit of the PI3K-α-selective inhibitor alpelisib in *PIK3CA*-mutated patients with HR + /HER2- advanced or metastatic BC [8, 9, 38], the vast majority of *PIK3CA* mutations were determined at the primary tumor (77%) rather than at the metastatic sites (22%) [39], whereas no matched tumors have been evaluated. Nonetheless, the presence of temporal tumor heterogeneity during metastatic progression should not be disregarded, since it could influence patient prognosis and drive treatment selection. Following the paradigm of other common BC biomarkers (i.e. ER, PR, HER2, PD-L1) and their discordance over time [40–42], the magnitude of discordance on *PIK3CA* mutational status between primary and metastatic lesions could have important clinical implications. The results of this meta-analysis indicate a fairly substantial change in the *PIK3CA*-mutational status—although at a lower level compared to immunohistochemistry-based biomarkers—further motivating metastatic biopsies, despite the anatomical, technical and analytical challenges [43]. On the other hand, one could argue that an approximately 10% discordance rate might be acceptable under certain circumstances and, as a result, PIK3CA-mutational analysis of the primary tumor could serve as a suitable option in patients where metastatic biopsies are deemed inappropriate or technically infeasibly.

Although the results of subgroup analyses based on metastatic sites are limited by the small study numbers, a numerically higher discordance rate in brain compared to liver lesions was observed. These findings should be confirmed by subsequent studies but the different discordance rates among different metastatic sites seem to be in accordance with emerging data on specific genomic alterations linked to specific organotropisms in breast cancer [44, 45].

The evaluation process of the *PIK3CA* mutational status could be influenced by several factors as the source of testing material and the detection method used. Regarding the former, the introduction of liquid biopsy and ctDNA analysis has gained interest as an appealing, non-invasive alternative method to tissue (re)biopsy [46]. Despite the fact that in most studies a benefit for PI3K inhibitors was demonstrated in ctDNA-detected *PIK3CA*-mutated patients [47], few studies have investigated the concordance of ctDNA- versus tissue-based approaches. These studies report a modest concordance (70–83%) between the two methods [48–50], indicating the risk of false negative result due to low- or non-tumor shedding, technical challenges and/or tumor heterogeneity. Of note, although the positive ctDNA *PIK3CA*-mutated patients received similar magnitude of benefit from alpelisib as for the tissue-based detection (HR = 0.55) in the SOLAR-1 study [50], negative ctDNA result did not preclude the presence of a *PIK3CA* mutation [51], imposing the analysis on tumor tissue and the need for obtaining a metastatic biopsy in patients with ctDNA-negative result and reflex testing in the primary tumor when biopsy from metastatic lesion is not feasible. Investigating the concordance between tissue-based and liquid biopsy-based *PIK3CA* analysis was beyond the scope of this systematic review and meta-analysis. Considering the mutation detection methods, the regulatory FDA approval of alpelisib included the use of *therascreen* PIK3CA RGQ PCR Kit, the FoundationOne® CDx and FoundationOne® Liquid CDx assays as companion diagnostics for the detection of *PIK3CA* mutational status. However, a post-hoc targeted NGS analysis of the SOLAR-1 tissue samples (initially tested with PCR-based assays aimed to detect 12 mutations in exons 7, 9, and 20) revealed that in 12% of patients with *PIK3CA*-altered status, a *PIK3CA* mutation was not previously detected by PCR and that these patients had a favorable outcome when treated with alpelisib [52]. Therefore, the analytical performance of the assays needs to be refined and standardized in order to reliably detect *PIK3CA* mutations. In our analysis, the majority of the studies included tissue samples tested with targeted NGS, without any further reported direct method comparison.

The present study suffers from limitations that need to be addressed. First, this is a study-level meta-analysis not including individual patient data that would enable a deeper analysis of patient subgroups, thus reflecting a substantial between-the-study heterogeneity. Second, substantial clinical differences in patient cohorts, treatment strategies, metastatic sites, detection methods used and determination of the *PIK3CA* mutational status among eligible studies were observed, thus reflecting a substantial statistical heterogeneity in almost all pooled analyses. In an effort to reduce the risk of bias due to between-study heterogeneity, we used random-effects model for pooled analyses. Furthermore, the small numbers of studies and patients resulted in limited number of tumor pairs for certain subgroups analyses including IHC-based subtypes and metastatic sites. Finally, no study included information on treatment with alpelisib or other PI3K inhibitors among eligible patients and no information about the effectiveness of PI3K inhibitors in discordant cases was reported.

In conclusion, this meta-analysis provides information on the overall discordance rates of *PIK3CA*-mutational status between primary and matched metastatic breast tumors, the direction of change as well as the impact of different subtypes and metastatic sites on discordance. Given the clinical benefit of PI3KCA inhibitors in *PIK3CA*-mutated metastatic BC, the analytical challenges of ctDNA testing and the observation that *PIK3CA* status could change in 1 out of 10 patients, our results indicate the need of obtaining metastatic biopsies for *PIK3CA*-mutation analysis but also the possibility of testing of the primary tumor, in case a re-biopsy deemed non-feasible.

## Supplementary Information

Below is the link to the electronic supplementary material.

Supplementary file1 (PDF 83 KB)

Supplementary file2 (PDF 286 KB)

## Data Availability

The datasets used and/or analysed during the current study are available from the corresponding author on reasonable request.

## Declarations

### Data sharing

The datasets used and/or analysed during the current study are available from the corresponding author on reasonable request.
