# Supplementary material for: Discordance of PIK3CA mutational status between primary and metastatic breast cancer: a systematic review and meta-analysis
Source: Breast Cancer Res Treat. 2023 Jul 1;201(2):161–9. doi: 10.1007/s10549-023-07010-1 (PMC10361863; doi:10.1007/s10549-023-07010-1)
Supplement: Supplementary file 1 — Supplementary file1 (PDF 83 KB) [file 10549_2023_7010_MOESM1_ESM.pdf]

Supplementary material to:

Discordance of PIK3CA mutational status between primary and metastatic breast cancer: A systematic review and meta-analysis

Justus Rosin[1*], Ella Svegrup[1*], Antonios Valachis[2#], Ioannis Zerdes[3,4#]

Authors' affiliations: 1. School of Medical Sciences, Örebro University, Örebro, Sweden; 2. Department of Oncology, Faculty of Medicine and Health, Örebro University Hospital, Örebro University, Örebro, Sweden; 3. Department of Oncology-Pathology, Karolinska Institutet, Stockholm, Sweden; 4. Breast Cancer Centre, Theme Cancer, Karolinska University Hospital, Stockholm, Sweden

* These authors contributed equally to the work

# These authors jointly supervised the work

Total number of supplementary-only material: 1 Supplementary Figure and Supplementary Methods

Supplementary Methods: Searching strategy in PubMed/Web of Science and Embase

A) PubMed/Web of Science

**Search terms**		**Items found**
**Population: Primary and metastatic breast cancer**		
1	(breast cancer*[Title/Abstract]) OR (breast malignan*[Title/Abstract]) OR (breast neoplas*[Title/Abstract]) OR (breast carcinoma*[Title/Abstract]) OR (breast tumo*[Title/Abstract])	363,248
2	(metasta*[Title/Abstract]) OR (primary[Title/Abstract]) OR (secondary[Title/Abstract]) OR (disseminated[Title/Abstract])	2,886,289
1 AND 2		154,702
**Exposure: PIK3CA**		
3	PIK3CA	6,967
Combined sets		
1 AND 2 AND 3		832

Searching strategy:

PIK3CA[Title/Abstract] AND ((breast cancer*[Title/Abstract]) OR (breast malignan*[Title/Abstract]) OR (breast neoplas*[Title/Abstract]) OR (breast carcinoma*[Title/Abstract]) OR (breast tumo*[Title/Abstract])) AND ((metasta*[Title/Abstract]) OR (primary[Title/Abstract]) OR (secondary[Title/Abstract]) OR (disseminated[Title/Abstract]))

B) Embase

No.	Query	Results
#7	#5 NOT #6	623
#6	#3 AND #4 AND ([conference abstract]/lim OR [conference paper]/lim OR [conference review]/lim)	1241
#5	#3 AND #4	1864
#4	pik3ca:ti,ab	14587
#3	#1 AND #2	172958
#2	metasta*:ti,ab OR primary:ti,ab OR secondary:ti,ab OR disseminated:ti,ab	3997986
#1	'breast cancer*':ti,ab OR 'breast malignan*':ti,ab OR 'breast neoplas*':ti,ab OR 'breast carcinoma*':ti,ab OR 'breast tumo*':ti,ab	510930

Supplementary Figure Legends

Supplementary Figure 1. Funnel plots assessing publication bias, for studies reporting *PIK3CA* mutational status pooled discordance rates. A. Overall discordance rate; B. Overall discordance rate from *PIK3CA*-mutated to *PIK3CA*-wild-type; C. Overall discordance rate from *PIK3CA*-wild-type to *PIK3CA*-mutated
