# Supplementary material for: Discordance of PIK3CA mutational status between primary and metastatic breast cancer: a systematic review and meta-analysis
Source: Breast Cancer Res Treat. 2023 Jul 1;201(2):161–9. doi: 10.1007/s10549-023-07010-1 (PMC10361863; doi:10.1007/s10549-023-07010-1)
Supplement: Supplementary file 2 — Supplementary file2 (PDF 286 KB) [file 10549_2023_7010_MOESM2_ESM.pdf]

Supplementary Figure 1. Funnel plots assessing publication bias, for studies reporting PIK3CA mutational status pooled discordance rates. A. Overall discordance rate; B. Overall discordance rate from PIK3CA-mutated to PIK3CA-wild-type; C. Overall discordance rate from PIK3CA-wild-type to PIK3CA-mutated

A

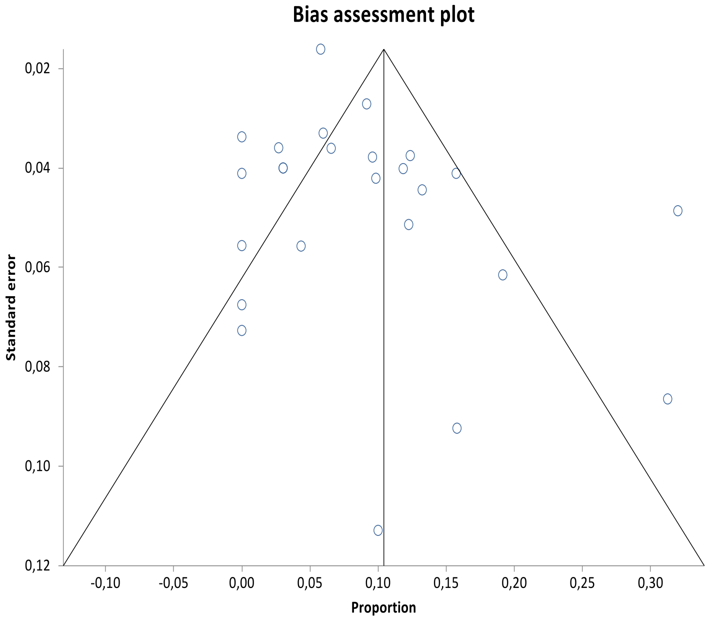

Begg-Mazumdar: Kendall's 0,217391 P = 0,1348

Egger: bias = 1,148938 (95% CI = -0,535455 to 2,833331) P = 0,1716

Harbord: bias = -0,790959 (92,5% CI = -2,818292 to 1,236375) P = 0,4742

B

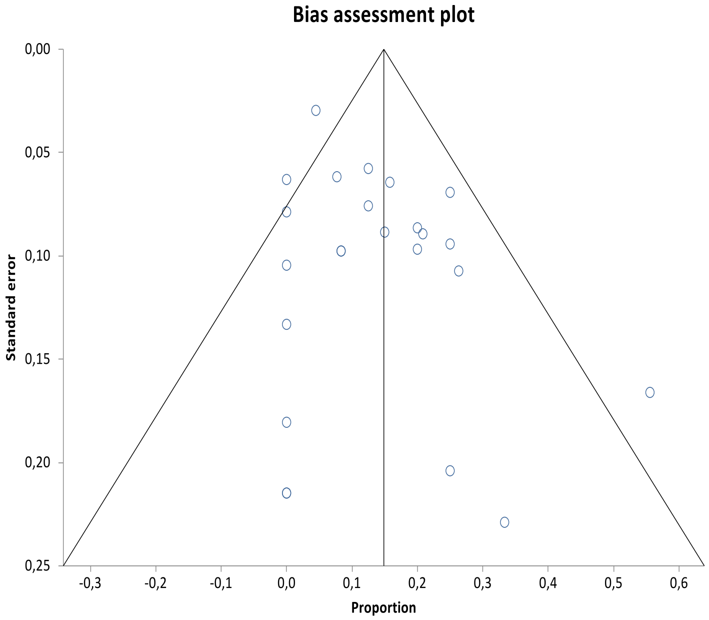

Begg-Mazumdar: Kendall's 0,094891 P = 0,5347

Egger: bias = 0,996692 (95% CI = -0,021999 to 2,015382) P = 0,0547

Harbord: bias = -0,013746 (92,5% CI = -1,235155 to 1,207664) P = 0,9834

C

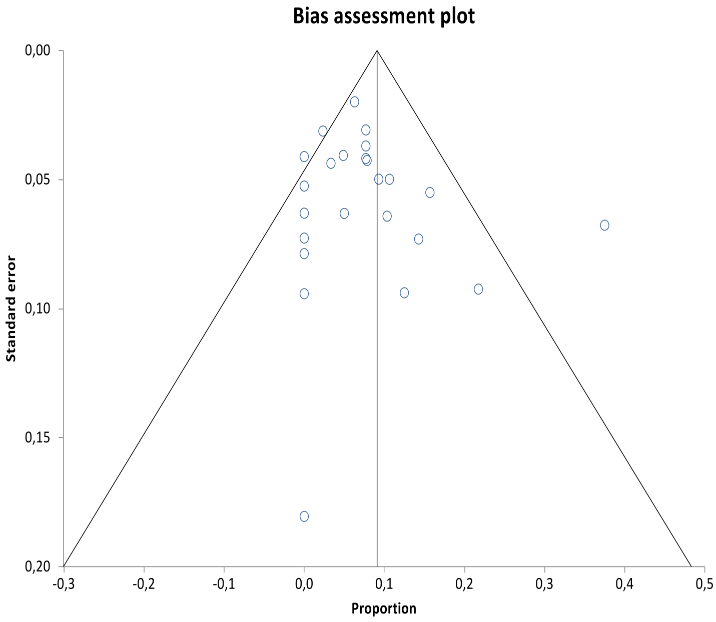

Begg-Mazumdar: Kendall's 0,166667 P = 0,2675

Egger: bias = 0,618025 (95% CI = -0,707182 to 1,943233) P = 0,344

Harbord: bias = -0,646081 (92,5% CI = -2,493965 to 1,201803) P = 0,5202
